# The role of intra- and interdialytic sodium balance and restriction in dialysis therapies

**DOI:** 10.3389/fmed.2023.1268319

**Published:** 2023-12-04

**Authors:** Susie Q. Lew, Gulay Asci, Paul A. Rootjes, Ercan Ok, Erik L. Penne, Ramin Sam, Antonios H. Tzamaloukas, Todd S. Ing, Jochen G. Raimann

**Affiliations:** ^1^Department of Medicine, George Washington University, Washington, DC, United States; ^2^Department of Nephrology, Ege University Medical School, Izmir, Türkiye; ^3^Department of Internal Medicine, Gelre Hospitals, Apeldoorn, Netherlands; ^4^Department of Nephrology, Northwest Clinics, Alkmaar, Netherlands; ^5^Division of Nephrology, Zuckerberg San Francisco General Hospital, University of California, San Francisco, San Francisco, CA, United States; ^6^Research Service, Raymond G. Murphy Veterans Affairs Medical Center, University of New Mexico School of Medicine, Albuquerque, NM, United States; ^7^Department of Medicine, Stritch School of Medicine, Loyola University Chicago, Maywood, IL, United States; ^8^Research Division, Renal Research Institute, New York City, NY, United States; ^9^Katz School of Science and Health at Yeshiva University, New York City, NY, United States

**Keywords:** sodium, chronic kidney disease, salt, dialysis, hypertension, extracellular volume, diet

## Abstract

The relationship between sodium, blood pressure and extracellular volume could not be more pronounced or complex than in a dialysis patient. We review the patients’ sources of sodium exposure in the form of dietary salt intake, medication administration, and the dialysis treatment itself. In addition, the roles dialysis modalities, hemodialysis types, and dialysis fluid sodium concentration have on blood pressure, intradialytic symptoms, and interdialytic weight gain affect patient outcomes are discussed. We review whether sodium restriction (reduced salt intake), alteration in dialysis fluid sodium concentration and the different dialysis types have any impact on blood pressure, intradialytic symptoms, and interdialytic weight gain.

## Introduction

Healthy individuals require no more than 50 mmol/day of dietary salt (i.e., sodium chloride) to remain in balance (1). The kidneys play a key role in the homeostasis of sodium, with some contribution from other organ systems such as the gastrointestinal tract and skin. Kidney failure disrupts sodium balance resulting in sodium and volume overload and subsequent elevated blood pressure (BP). Therefore, rigorous restriction of salt intake continues to be recommended for patients with reduced to no kidney function.

In chronic kidney disease (CKD) patients, and particularly those receiving renal replacement therapy, there exists a well-reported relationship between extracellular fluid volume (ECV) and sodium balance. Dialysis patients gain sodium from dietary, medicinal, and dialytic sources (usually through diffusion) and lose sodium mainly through dialysis (mainly through ultrafiltration), insignificantly via kidneys in those with residual kidney function, and from other potential organ systems such as the gastrointestinal tract and skin. Higher sodium intake results in thirst that is subsequently satisfied by water ingestion, which leads to an increase in ECV. The increase in ECV stimulates natriuresis in kidneys to retract ECV to normal levels within hours and days (pressure natriuresis) (2). Therefore, kidney function is imperative for maintaining the ECV by changing daily sodium excretion. In patients with diminished kidney function, the capacity of the kidney to excrete sodium decreases, which causes sensitivity to sodium and an increase in BP (3). Sodium intake, not water, primarily defines volume. Volume overload causes hypertension. Consequently, ECV increase/volume overload is the primary factor in the pathogenesis of hypertension in patients with end-stage kidney disease (ESKD) (4).

Hypertension and/or volume overload occur commonly and have been associated with several deleterious outcomes such as left ventricular (LV) hypertrophy, LV systolic and diastolic dysfunction, cardiac enlargement, cardiac failure, ischemic heart disease, myocardial infarction, sudden cardiac death, stroke, and cardiovascular and overall mortality in dialysis patients (5–16). In a prospective study including 176,790 prevalent hemodialysis (HD) patients, it was shown that 14% of the patients required hospital admission for one or more episodes of fluid overload, heart failure or pulmonary edema necessitating urgent fluid removal during 2.5 years of follow-up (17).

Although volume overload (sodium retention) is known to be the main pathogenetic mechanism of hypertension, most patients are still prescribed several antihypertensive medications to control BP, notably often failing optimal control. In a cohort of 2,535 HD patients, the frequency of hypertension defined as systolic BP >150 mmHg or diastolic BP >85 mmHg or the use of antihypertensive medications was reported as 86% (18). Data from the Dialysis Outcomes and Practice Patterns Study (DOPPS) show that the prevalence of hypertension varies between 72.7% in the UK and 90.3% in the US (19).

With well-established associations, sodium, volume overload and hypertension are well established predictors of hospitalizations and death. In those with minimal to no residual kidney function, both diet and renal replacement therapy represent the most powerful means to control sodium, volume, and their consequences in the dialysis patient. In this report we discuss dietary and dialytic sodium mass balance and the effects of their restriction on outcomes in this population.

## Sodium in the diet

Recommended diets in developed countries have been suggested to contain 80–100 mmol of sodium per day, but many individuals consume substantially more than that (200–300 mmol per day) (1). Other estimates suggest that a Western-type diet contains around 12 g of salt per day (comprising 4.8 g or 200 mmol of sodium) (20), while generally sodium intake should be less than 2,300 mg per day or 5.8 g of salt (or 100 mmol) for individuals above the age of 14 years (21). The World Health Organization (WHO) recommends a salt intake of 5 g per day (approximately 2 g of sodium) for the prevention of cardiovascular diseases (22). The American Heart Association (AHA), the American College of Cardiology (ACC) and the Heart Failure Society of America (HFSA) guidelines for the management of heart failure recommend a sodium intake of less than 2.3 g per day to improve the general cardiovascular health in patients with heart failure, although there are notably no trials to support this level of restriction (23).

Individuals living in western societies do not have much choice regarding salt intake, because most dietary sodium (70–80%) comes from processed food or salty foods such as snacks, sauces, pickles, cheese, or bread, among others rather than salt added while cooking or eating. Therefore, it is not possible to talk about a salt-restricted diet unless sodium concentrations in processed food are reduced or people stop consuming all processed and cooked food. Since sodium is commonly complexed with phosphorus in food additives, it is not surprising that along with total sodium intake, additives in the processed foods also substantially increase total phosphorus content (10–50% of total phosphorus intake per day). Therefore, the impact of this excessive additive intake on subjects with CKD may be associated with much worse clinical outcomes. Consequently, and as outlined above, sodium- and phosphate-based food additives in processed foods represent large barriers in restriction of dietary sodium and phosphorus in patients with CKD.

All major nephrological guidelines recommend sodium restriction for the CKD population. Relying on data from both the general population and CKD patients, the KDIGO guideline for the management of BP in CKD indicates that a salt-restricted diet results in short-term reductions in BP and in a reduction in the need for antihypertensive medications. For CKD patients with high BP, in line with the general population, it is recommended to consume less than 2 g of sodium per day.

## The effects of salt restriction in the dialysis population

A lack of data from randomized trials on the effect of a sodium-restricted diet on cardiovascular disease is noted. In conclusion, the KDIGO guideline recommends a sodium intake of less than 2 g per day or less than 5 g of sodium chloride per day in CKD patients with high blood pressure (24). The KDOQI guideline on nutrition in CKD emphasizes, as in the KDIGO guideline described above, that the direct evidence for a sodium-restricted diet in the CKD population is not strong. Unlike the KDIGO guideline, the KDOQI guideline provides recommendations for different subpopulations within the spectrum of CKD patients. With regards to BP management of patients with CKD stage 3–5, CKD stage 5D and post-transplant patients, a sodium intake of less than 2.3 g per day is recommended to optimize BP and volume status. With regards to the treatment of proteinuria in CKD stage 3–5, a sodium intake of less than 2.3 g per day is also recommended in addition to the available pharmacological interventions. With regards to dry body weight, for patients with stage 3 to 5D CKD, a sodium-restricted diet is considered an additional lifestyle modification strategy to achieve better volume control (25).

For the dialysis population the case is most impressively made by reports from two centers practicing strict volume control strategy (Tassin in France and Ege University in Izmir, Türkiye). These centers report the frequency of patients with BP less than 140/90 mmHg as 98 and 96% without using antihypertensive medication. These numbers are in sharp contrast with the rest of the world, suggesting that the differences are related to the treatment methods (26, 27).

In the late 1970s, the Tassin group from France practiced long HD sessions together with dietary salt restriction and extracellular volume (ECV) reduction to treat hypertension instead of using antihypertensive drugs. In their first report in 1983, the center reported an overall 10-year survival rate of 85% (28). Their volume control regimen included salt restriction (4–5 g per day), controlled ultrafiltration (UF) during long HD sessions to achieve normal post-HD and pre-HD BP, and gradual cessation of antihypertensive drugs (29, 30). In another report including 876 HD patients who were followed up to 20 years, 90% of the population had high BP at the initiation of dialysis (31). During follow-up with a mean of 23.8 h HD per week, the mean interdialytic weight gain (IDWG) was 1.6 ± 0.4 kg, reflecting successful dietary salt restriction. Post-HD body weight was reduced by 2–3 kg by UF within the first month, yielding a decrease in BP. All antihypertensive medications were stopped within the first 2 months. In the second month body weight remained stable, but BP continued to decrease. Three months later, only less than 5% of the patients needed antihypertensive medications.

Then, body weight increased by several kilogram, but BP continued to come down gradually between the 3rd and 12th month, suggesting an anabolic weight gain with the increase of muscle and fat mass due to improved appetite (32). Progressive and slow BP decrease observed between the 3rd and 12th month in the absence of further ECV reduction was explained by delayed regression of peripheral resistance following reduction of ECV overload which is called the “lag phenomenon,” similar to that seen during treatment of primary hypertension with diuretics (31–33). Normal BP was achieved in 98% of the patients in the 6th month of follow-up.

With the combination of extended hours HD and strict volume control, they achieved excellent survival rates, which were much better than US, Japan, and Europe. In a study comparing the survival rate between patients in Tassin and HD patients from other regions with regards to fluid overload status assessed by multi-frequency bioimpedance analysis (34), the survival rate in euvolemic standard HD patients was close to the percentage in Tassin, while those from other regions that were fluid overloaded had markedly lower survival rates. These data underline the significant role of volume and BP control in the excellent survival rates reported from Tassin, although the survival benefit with longer HD sessions should not be ignored (35).

At Ege University “strict volume control” as a strategy was introduced in 1993. Prior to its implementation, 65% of patients were on antihypertensive medications, IDWG was over 3 kg, heart failure, intradialytic hypotension, and cramps were common, some patients were diagnosed as having developed uremic cardiomyopathy, and many patients frequently requested to stop their session earlier due to hypotension and cramps in the last hour of dialysis. The strict volume control strategy consisted of (a) re-emphasis on salt restriction (4–5 g per day), (b) stopping all antihypertensive medications, (c) intensified UF during standard HD sessions (three times a week for 4–5 h, dialysis fluid sodium 138 mmol/L), and (d) isolated UF sessions as needed.

As many patients commonly interpreted salt restriction as “no added salt,” its importance was explained to the patients and family members. Repeated instructions by doctors and/or nurses, written or oral clarification on the meaning of salt restriction and interviews by a dietician were all necessary to change the patients’ attitude. Based on the analysis of their food consumption, mean dietary salt intake was estimated to be around 4–5 g/day. It was explained by the dialysis team that water restriction alone is ineffective without salt restriction. Patients were advised to drink according to their thirst. In addition, patients and families were advised to restrict consumption of processed food with high sodium content and to consume salt-free bread.

Dry weight reduction was continued until BP became lower than 140/90 mmHg and cardiothoracic index (CTI) on chest x-ray was below 0.50. If BP remained >140/90 mmHg, but there was a doubt whether euvolemia was reached because CTI was close to normal (≤0.50), a 25 mg dose of captopril was given to evaluate the renin-dependency of the high BP on a non-dialysis day. Otherwise, UF continued until the target BP was reached. If too much weight was gained, an extra isolated UF session was added.

With this strategy, in a series of 218 HD patients, the mean BP decreased from 150/89 to 121/75 mmHg at the end of the observation period, with a mean of 47 months (27). Only 4% of patients needed antihypertensive medication. The mean CTI also dropped from 0.50 to 0.46, and IDWG decreased from 1.4 to 0.9 kg per day. The mortality rate was 68.2 per 1,000-patient-year, better than in most published series. Cardiomegaly (CTI >0.48) despite normal BP, had a strong negative impact on survival; patients with CTI > 0.48 showed mortality rate 3.8 times higher than those with CTI < 0.48. With this strategy, the frequency of intradialytic hypotensive episodes decreased from 22 to 7% (36). The volume control strategy also resulted in regression of LV hypertrophy (37), elimination of intradialytic paradoxical hypertension (38), successful treatment of patients with markedly low LV ejection fraction (39) and with valvular insufficiencies (mitral and tricuspid) in standard HD patients (40).

This strategy, first implemented at Ege University, Izmir, has been adopted by other nephrologists in the city, and then gradually spread to almost all regions of Turkey. Supporting this approach, the prevalence of hypertension (systolic BP >140 mmHg and/or diastolic BP >90 mmHg) was found to be 16% in 782 prevalent HD patients, who were the participants of a multicenter randomized controlled trial (RCT) involving 10 for-profit dialysis clinics located in different regions of the country (41). The mean systolic BP was 128 ± 15 mmHg, and the proportion of patients using antihypertensive medications was 13.0%. In 704 prevalent HD patients during a follow-up of 4 years, the frequency of hospitalization due to fluid overload/congestive heart failure/pulmonary edema was 7 per 1,000-patient-years in Izmir (unpublished data). This number was reported as 137 per 1,000-patient-years in the US (17).

These results demonstrate that volume control strategy can be conducted on a national level when both physicians and nurses are committed to the belief that normal BP can be achieved by dietary salt restriction and insistent UF without use of antihypertensive medications in standard HD patients. One might argue that the successful volume and BP control achieved in the Turkish case may be mainly attributed to the Mediterranean diet. However, in a national study in 1970 subjects, the daily average salt intake was found to be 18 g (42).

These results prove that satisfactory BP and volume control can be achieved by reducing post-HD body weight progressively and strict dietary salt restriction in standard HD patients. The major determinant of success in restricting dietary salt intake in dialysis clinics is the staff: How much they are dedicated, and how much effort they must put into educating the patients and their families. The treating physicians are to be convinced first, then nurses, and then finally, patients and their families. It should be noted that nurses are in many cases the key to convincing both patients and their families.

## The importance of dietary sodium restriction in dialysis patients

### Sources of dietary sodium

Knowing the patient’s sodium intake allows for constructive nutritional advice rather than speaking to patients about a hypothetical diet. Sodium intake generally can be estimated using dietary recalls or food frequency questionnaires (FFQs) such as the Derby Salt Questionnaire (DSQ) (43), the Royal Free Sodium Questionnaire (RFSQ) (44), or the Scored Sodium Questionnaire (SSQ) (45). Using such dietary recalls, Clark-Cutaia et al. found that younger patients encountered more difficulty adhering to the dialysis diet and for restricting sodium intake (46). Younger patients had a higher median sodium intake and higher average adjusted interdialytic weight gain. Additionally, female patients reported more problems managing their diet. In another study, investigators found that men had higher estimated dietary sodium intake (47). In the same study, the investigators not only confirmed that younger patients but also those aged over 75 years had higher sodium intake. In a study by Amalia and Davenport (44), the authors found in peritoneal dialysis (PD) patients that median estimated dietary sodium intake was 2.39 g sodium per day by DSQ and 2.11 g sodium per day by RFSQ. Patients younger than 53 years had a higher sodium intake compared to those older than 76 years [RFSQ 105.4 (73–129) versus 96 (71–116) mmol per day; *p* < 0.05].

Consequent to recognition of the adverse effects of high sodium diets in individuals, many asked how to efficiently lower intake on a population level. Various agencies and institutions, such as the Food and Drug Administration (FDA) released guidance which aimed at recommending the reduction of salt in food that people residing in the US consume at restaurants, school cafeterias, food trucks, or when eating processed, packaged, and prepared foods at home. These recommendations seek to reduce the average daily sodium intake by encouraging food manufacturers, restaurants, and food service companies to scale back their use of salt. Initiatives such as the National Salt and Sugar Reduction initiative, a partnership of organizations and health authorities from across the country, convened by the New York City Department of Health also aim to reduce, next to sugar, the levels of sodium in food and beverages. Also, in the European Union similar policy recommendations to reduce sodium intake have been defined, ranging from voluntary actions (e.g., targeted sodium reduction in all food products) to legally binding obligations (e.g., a maximum level of sodium in bread in some European countries). A recent meta-analysis suggests that generally multi-component interventional approaches that include a structure nature (e.g., food product reformulation, food procurement policy in specific settings) appear more effective than single-component initiatives such as information campaigns alone (48).

These initiatives partly address the large problem with adhering to low-sodium recommendations caused by food that was not self-prepared. It has been well established that sodium not only is in table salt but can also be found in food that was not self-prepared, particularly prominent in processed food products. Table 1 highlights some high sodium-level containing foods. Consequent to its ubiquitous presence, adhering to a 2 g sodium diet requires diligence to review food labels, patient interest and research into the lesser-known sodium sources, and a disciplined selection of appropriate food items containing low sodium. Particularly, avoidance if possible or at least careful selection of preserved food, which traditionally contains high levels of sodium, has repeatedly been suggested and appears to be central to a successful dietary sodium restriction. Further, recognizing underappreciated sources of sodium, such as bread, must be considered as well (49).

**Table 1:** Sodium content in selected foods to highlight high sodium content.

Food	Portion size	Sodium (mg)
Bacon, Turkey cooked	3 medium slices	754
Beans, black, canned	½ cup	511
Beans, baked, canned	½ cup	436
Biscuit	1 medium	275
Bologna	1 slice	272
Bread, whole wheat	1 slice	132
Cereal, Corn Flakes	1 cup	202
Cheese, American	1 oz	461
Chicken, light meat, coated, fried	3 oz	374
Clams, moisture cooked	19 small	897
Cornbread	2.1 oz	463
Crab, blue, moisture, cooked	3 oz	336
Fish, flounder, cooked	3 oz	309
Hot dog, pork	1 link	620
Macaroni and cheese	1 cup	477
Muffin, corn	1 medium	723
Pickle, dill	1 large	1,181
Pie, pumpkin, frozen	1/6 of 8”	450
Pita bread, white	1 large	322
Pizza, cheese, meat	1/8 of 12”	769
Potato, French fried, frozen	10 strips	485
Sausage, pork	2 links	360
Shrimp, moisture cooked	3 oz	805
Soup, chicken noodle	1 cup	866
Tuna, canned in water	3 oz	287

Adopted from American Association of Kidney Patient Nutrition Counter: A reference for Kidney Patients. Aakp.org.

Examples of underappreciated sources of sodium include oral medicines, some of which have added sodium to aid absorption. As an example, effervescent tablets use sodium bicarbonate to make them fizz. Other medications use sodium compounds to make medications soluble and dispersible. Table 2 shows sodium content in frequently prescribed medications to dialysis patients.

**Table 2:** Sodium content in commonly prescribed medications used by patients with kidney failure.

Medication class	Medication	Dose size	Sodium-based compound	Sodium (mg)
Anemia medications				
	Epoetin alfa	Single dose: 1 mL vial	Sodium chloride Sodium citrate	5.9 5.8
		Multi-dose: 2 mL vial	Sodium chloride Sodium citrate	8.2 1.3
	Darbepoetin alfa	1 mL vial	Sodium chloride Sodium phosphate dibasic anhydrous Sodium phosphate monobasic monohydrate	8.18 0.66 2.12
	Methoxy polyethylene glycol-epoetin beta	0.3 mL	Sodium phosphate monobasic monohydrate Sodium sulfate	0.414 1.704
Antiacids				
	Famotidine	20 or 40 mg tablet	No sodium	
		Oral suspension	Added preservatives: Sodium benzoate 0.1%, Sodium methylparaben 0.1%, sodium propylparaben 0.02%	
	Ranitidine	150 mg 300 mg 25 EFFER syrup (per mL)	none Croscarmellose sodium Monosodium citrate anhydrous Sodium bicarbonate Sodium benzoate Dibasic sodium phosphate Saccharin sodium Sodium chloride	Not specified Total sodium 30.52 Not specified
	Pantoprazole sodium	20 or 40 mg Delayed-Release oral suspension	Sodium carbonate, sodium lauryl sulfate Same as above	Not specified
	Esomeprazole	Delayed –release capsule 20 or 40 mg	Sodium hydroxide	Amount unknown
	Omeprazole	20 or 40 mg Powder for oral suspension of 20 or 40 mg	Sodium bicarbonate Croscarmellose sodium Sodium stearyl fumarate Sodium bicarbonate	1,100 Not specified 1,680
Antihypertensive medications				
	Nifedipine XL		Sodium chloride	Not specified
	Amlodipine		Sodium starch	Not specified
	Diltiazem		Sodium starch glycolate	Not specified
	Labetalol		Sodium benzoate	Not specified
	Lisinopril		None	
	Losartan		None	
	Metoprolol succinate		Sodium stearyl fumarate	Not specified
	Metoprolol tartrate injection	Per 5 mL ampules	Sodium chloride	45
	Metoprolol tartrate tablet		Sodium starch	Not specified
	Carvedilol		None	
	Hydralazine injection		Sodium hydroxide	Not specified
	Hydralazine		None	
	Clonidine tablet		None	
	Minoxidil		None	
Bicarbonate supplement				
	Sodium bicarbonate	650 mg	Sodium bicarbonate	178
Diuretics	Furosemide injection		Sodium hydroxide Sodium chloride	Not specified
	Furosemide tablet		None	
	Bumetanide injection	Per mL	Sodium chloride Edetate disodium	8.5 0.1
	Bumetanide tablet		None	
Laxatives	Docusate sodium	100 mg		5
	Polyethylene glycol		None	
	Lactulose		None	
	Linaclotide		None	
	Lubiprostone		None	
Phosphate binder	Sevelamer		Sodium chloride	Not specified
	Lanthanum carbonate		None	
	Ferric citrate		None	
	Calcium acetate		None	
Potassium binder	Sodium polystyrene sulfonate	Per gm	Sodium polystyrene sulfonate	100
	Sodium zirconium cyclosilicate	5 gm	Sodium zirconium cyclosilicate	400
	Patiromer sorbitex calcium		None	
Vitamins				
	Calcitriol injection	1 mL	Sodium ascorbate	2.5
	Calcitriol tablet		None	
	Paricalcitol injection		None	
	Paricalcitol capsule		None	
	Doxercalciferol injection	Per mL	Sodium chloride Sodium ascorbate Sodium phosphate dibasic Sodium phosphate monobasic Disodium edetate	1.5 10 7.6 1.8 1.1
	Doxercalciferol capsule		None	

Sources from accessdata.fda.gov.

Sodium bicarbonate represents an example of an oral medication containing sodium. Nephrologists frequently prescribe 1 tablet (650 mg sodium bicarbonate) three times daily which contains 534 mg of sodium or 2 tablets three times daily which translates into 1,068 mg of sodium, a non-trivial amount of sodium in a diet restricted to 2,000 mg per day.

### The body’s response to sodium restriction or depletion

In non-dialysis patients, the kidney adaptively retains sodium chloride and the individual increases sodium appetite in response to salt restriction or sodium depletion to restore sodium balance. Kidney sodium retention and an increase in sodium appetite can be maladaptive and sustain pathophysiologic conditions such as salt-sensitive hypertension and chronic heart failure (50). The mineralocorticoid aldosterone plays a central role in both the increase in renal salt reabsorption and sodium appetite (50). Fu and Vallon hypothesize that aldosterone activates similar signaling and effector mechanisms in the kidney and brain, including the mineralocorticoid receptor, the serum- and glucocorticoid-induced kinase SGK1, the ubiquitin ligase NEDD4-2, and the epithelial sodium channel ENaC. ENaC also mediates the gustatory salt sensing in the tongue, which is required for the manifestation of increased salt intake (50). The effects of aldosterone on both the brain and kidney synergize with the effects of angiotensin II.

In dialysis patients this fueled the hypothesis by Leshem and Rudoy (51) who studied the preference of salt content in soup by dialysis patients before the dialysis treatment and 24 h thereafter. Hypertensive dialysis patients increased their preference for salt after treatment like normotensives. The authors suggest that humans may respond to reduction in total body sodium with a delayed increase in preference for salt.

Studies in PD patients, comparing them to a control cohort, and HD and kidney transplant patients, demonstrated a higher salt appetite according to their perceived taste intensity to varying concentration of sodium in a salt solution (52). Salt appetite has also been described in patients with iron deficiency, a diagnosis that patients with ESKD frequently experience, according to a case report from the 1980s (53).

### The body’s response to a high sodium intake

One important new dimension in the concepts of body sodium compartments and adverse effects of high sodium intake in both healthy individuals and subjects with CKD consists of the discovery that the osmotically inactive sodium compartment interacts with the osmotically active compartment in the extracellular compartment under conditions of high or low sodium intake (54, 55). Two long-term (7 days or longer) studies of varying intake of sodium documented retention of sodium in the absence of water gain at high sodium intake (56, 57). Heer and co-investigators (56) observed an increase in the plasma volume without an increase in body water at a high sodium intake. Rossitto et al. (58) reported a systemic isotonic shift of water from the intracellular into the extracellular compartment at high sodium intake.

The osmotically inactive sodium compartment which interacts with the osmotically active compartment exists in polyanionic proteoglycans found in skin, cartilage, bones, muscles, and endothelial surface layers (59, 60). Glycosaminoglycan, a proteoglycan, has been shown to be a major compound binding sodium non-osmotically (61). Glycosaminoglycan structural abnormalities in hereditary and acquired diseases affect the deposition of sodium in the non-osmotically active compartment (59).

Sodium uptake by the osmotically inactive compartment at high sodium intake has adverse structural consequences. Changes in sodium content of the osmotically inactive compartment are detected by sodium magnetic resonance imaging using ^23^Na (62). Using this technology, Kopp and co-investigators (62) observed increasing with age sodium content in muscle of men, but not of women, and in skin, while the water content of muscle of men did not increase; sodium and water content of skin increased in both genders, but less in women; and sodium content was higher in subjects with refractory hypertension and comparable age.

Increases in sodium content at high sodium intake have several adverse effects in all stages of CKD. Yu and co-investigators (63) documented high sodium intake-induced elevated BP, left ventricular and renal hypertrophy, interstitial fibrosis in the left ventricle and kidneys of both spontaneously hypertensive and normotensive rats; a documented overexpression of the cytokine transforming growth factor beta 1 (TGF-β1) was proposed as the putative mechanism of the cardiac and renal tissue changes. In patients with CKD, Schneider, and co-investigators (64) reported a statistical association between skin sodium concentration and left ventricular hypertrophy which was stronger than the associations of ventricular hypertrophy with hypertension or volume overload. Oppelaar and Vogt (65) reviewed the adverse effects of sodium deposition at high sodium intake in the renal osmotically inactive compartment, which they attributed to inflammation and development of fibrosis and suggested that improvement in the understanding of these mechanisms may lead to new methods of treatment. Ito and co-authors reported that binding and subsequent release of sodium to tissue proteoglycans regulates local tonicity and activates the tonicity-responsive enhancer-binding protein (TonEBP), which has a role in inducing inflammation of organs (66). Inflammation is an important contributor to adverse outcomes of high sodium intake. In a study that recruited many subjects, Wenstedt et al. (67) documented an independent statistical association between high sodium intake and elevated circulating granulocyte concentrations, and adverse cardiovascular and renal outcomes. Akbari and McIntyre (68) reported that excess Na tissue accumulation is associated with declining renal function in all stages of CKD, hypertension, inflammation, and cardiovascular dysfunction. The association of tissue sodium content with inflammation and adverse outcomes is found also in patients on dialysis. Sahinoz et al. (69) reported that patients on either HD or PD have higher sodium content in skin than subjects without kidney disease and that plasma levels of IL-6 and high-sensitivity CRP correlate with muscle and skin sodium content in these patients. High sodium content in the peritoneal membrane has adverse structural effects. Sun and coinvestigators (70) reported that high sodium intake in mice subjected to subtotal nephrectomy resulted in structural changes in the peritoneum and higher peritoneal solute transport state, although the peritoneum had not been exposed to dialysis fluid.

### The effects of sodium restriction on the body

As a result of the evidence from controlled clinical studies, the positive effect of consuming less salt on BP is well known not only in patients with CKD but also in the general population. In a meta-analysis including 133 trials with more than 12,000 subjects aged over 18 years without CKD and heart failure, it was shown that there was a dose-dependent correlation between salt restriction and decrease in BP levels (71). The mean reduction of 24-h urinary sodium excretion, systolic and diastolic BP in the low sodium consumption group were 130 mmol (*p* < 0.001), 4.26 mmHg (*p* < 0.001), and 2.07 mmHg (*p* < 0.001), respectively. For the same reduction in urinary sodium level, there was greater systolic BP reduction in older people, non-white people, and those with higher baseline systolic BP levels. In addition, a greater dose–response association between sodium reduction and lower BP was found in trials with study duration of more than 2 weeks compared to the trials of shorter duration. Therefore, it seems that maintaining a low-salt diet in the long run eventually results in a more pronounced reduction in BP. A low-salt diet has been associated with smaller shifts of body fluids from the interstitial into the intravascular compartment, a decreased need for use of antihypertensive medications, a lower production of asymmetric dimethylarginine (ADMA), a lower generation of TGFβ-mRNA, and lower activation of mitogen-activated protein kinases (MAPK) (72). For patients with no or minimal kidney function receiving chronic maintenance renal replacement therapy the situation is more complicated.

Increased salt intake has been shown to be independently associated with high pre-dialysis systolic BP and mortality in HD patients. Conversely, sodium restriction in dialysis patients has been reported to help manage BP and volume overload, subsequently preventing left ventricular hypertrophy, and decreasing mortality (73).

In the HEMO Study, which included 1,800 patients on HD, it was reported that a dietary sodium content of more than 2.5 g per day was associated with increased risk of death (74). Normalization of volume overload holds the key to control BP and to reduce cardiovascular events; as indicated by the recent guideline, dietary salt restriction should be below 5–6 g per day and IDWG should not exceed 0.8 kg per day (75).

### The sociological and psychological dimension of sodium restriction

From a social perspective, the positive effect of lifetime salt restriction looks obvious. In a long-term intervention study of the effect of reduction in salt intake in one of two similar villages in Portugal, a difference in BP between two villages was detectable over the course of 2 years (76). The most relevant information on the effect of lifelong low salt intake on BP was recorded in societies isolated from influences of civilization (77). Moreover, the effect of salt restriction on BP of a short period of time in the newborn persists into adolescence (78). As a public health problem, high salt intake has been related to not only hypertension but also other undesirable effects including stomach carcinoma, stroke, LV hypertrophy, microalbuminuria and hypercalciuria.

Furthermore, low socioeconomic status may promote excess intake of relatively inexpensive processed and fast foods enriched with phosphate and salt. It was reported that people of low socioeconomic status likely consume more sodium than people of high socioeconomic status (79). It shows the importance of global and regional targets to reduce sodium intake at the population level and interventions aimed at reducing socioeconomic disparities in diet quality and health. Moreover, in a study of Japanese workers, it was shown that both years of education and household income significantly affected not only salt intake but also the risk of hypertension. Those subjects with higher levels of education or income had a lower risk to become hypertensive (80).

Consumption of salted food represents a social habit with characteristics of an “addiction” (81, 82). It takes weeks to adapt to a lower “salt level,” during which time low-salt food is experienced as very “tasteless.” Patients are therefore unwilling to accept such a salt-restricted diet and would prefer medicine. Although salt restriction may cause appetite loss at the beginning, it improves salt sensation (83).

It takes a lot of effort and time for a doctor to convince patients to reduce dietary salt intake. The time requested for this adaptation may be several weeks to months. First, the healthcare partners need to be convinced of the feasibility of sodium intake reduction in their population. Then a coordinated program must be initiated and monitored. This program must be enhanced by organizing discussions including the patient and his/her family. Consequently, salt restriction can be possible resulting in benefits for the well-being of the patients. It has been shown that patients’ education for salt-restricted diet was associated with decrease in IDWG by 30% in HD patients (84).

Some dialysis centers offer “normal meals” to the patients during dialysis sessions. This approach will eventually lead to an adaptation to taste for salt and may then translate to a general embracement of a less-salt inclined palate.

Some experts in the field have also suggested that psychological techniques of empowering patients to take control over their behavior and adhere to recommendations, such as Motivational Interviewing, could be successful in aiding the problem (85, 86).

Commercial concerns may be an obstacle to the implementation of salt restriction. It is very difficult to implement and maintain a successful salt reduction program without the help of the food industry. A salt intake reduction program including salt-awareness campaigns, collaboration with food industry, labeling and reducing the amount of salt in products has been successfully implemented in only a few countries (Finland, Japan, Portugal, and the United Kingdom) (87). In the 1970s, these salt restriction strategies were associated not only with improved survival but also with lower costs for the countries. To pursue the goal to adequately control hypertension globally at low cost, an important requirement for low-income countries, it is essential to take effective measures to limit dietary salt intake and raise awareness of the harmful effects of salt.

## Sodium in renal replacement therapy

### Sources of sodium

#### Sources for sodium addition and removal in hemodialysis

Functioning kidneys remove sodium and water 24 h per day, 7 days per week. As such fluid and salt removal occurs slowly and consistently. With dialysis one tries to remove 2–3 liters of water containing 280–420 mEq of sodium (assuming sodium concentration of 140 mEq/L) over 3–4 h. These fast ultrafiltration rates result in cramps, hypotension, fatigue, dizziness, nausea, among other symptoms during dialysis, limiting fluid removal. In addition, it leaves dialysis patients chronically volume overloaded, sodium overloaded and hypertensive. Thus, it is intuitive that longer and more frequent dialysis sessions go a long way to solve this issue. The problem is that in many areas of the world more frequent dialysis is not systemically possible in a widespread basis and also one often encounters lot of patient resistance to longer treatments from patients who often experience unwelcome side effects from dialysis and are used to short dialysis treatments.

Since we do not want to induce hypo or hypernatremia with dialysis treatments, the sodium concentration in the dialysis fluid is similar to that of the blood. As such sodium removal/gain with diffusion during dialysis is minimal. Of course, it depends on the sodium bath used with dialysis and the patient’s serum sodium concentration. However, we do need to remove sodium with dialysis as patients eat salt between dialysis treatments and often are unable to remove the extra salt through their limited kidney function or for instance the gastrointestinal tract. The extra sodium is removed through ultrafiltration as the fluid removed with dialysis has a sodium concentration similar to the blood serum sodium concentration.

Priming the dialysis circuit requires approximately 300 mL of 0.9% saline to remove air and debris and soften the dialyzer membrane. Depending on the dialyzer size the priming volume ranges from 60 to 120 mL. The arterial and venous blood tubing volume are 94 and 62 mL, respectively. This volume of 0.9% saline is infused into the patient (88). At the end of dialysis, blood must be returned to the patient in a closed system. Approximately 500 mL of 0.9% saline is administered via the arterial blood tubing to accomplish this. An additional 10 mL saline is used to flush the arterial access. The dialysis treatment takes this into account by adding 500 mL UF to the prescribed UF.

BP remains stable during dialysis if extravascular volume replaces intravascular volume lost by UF. BP decreases when ultrafiltration exceeds intravascular volume replacement from extravascular volume sources. Hypotension and cramps result in discontinuation of the ultrafiltration and administration of saline by the dialysis staff. Usually, a few hundred milliliters of 0.9% saline achieves the purpose. Historically, 10–15 ml of 23.4% hypertonic saline was administered. This practice has ceased due to complications associated with administering sodium over a short period of time, namely, hypernatremia, thirst, hypertension, and volume overload.

Albumin infusion represents another means of treating hypotension. For example, a 20% solution, with 96% or more human albumin contains 130–160 mmol of sodium in a volume of 1 L. During dialysis, 50 mL of 20% albumin are infused for hypotension; this contains 6.5–8.0 mmol of sodium. Albumin infusion provides predominantly colloidal but also some crystalloid properties.

During HD sodium fluxes across the dialyzer membrane along a concentration gradient from a higher to lower concentration to reach equilibrium. Patients can either gain or lose sodium. Sodium gain leads to maintaining or increasing BP, while sodium loss may result in hypotension. Many studies have documented sodium gain during dialysis when the dialysis fluid sodium concentration is higher than plasma sodium (89–91).

Hyponatremia occurs more frequently in dialysis patients than hypernatremia. Dialysis patients develop hyponatremia because of excessive water intake. It has been suggested that pre-HD hyponatremia is more than just water excess; hyponatremia has been associated with loss of muscle mass and strength, increased C-reactive protein (CRP) levels and reduced serum albumin levels, suggesting that hyponatremia is associated with inflammation, loss of muscle mass and increasing frailty (92).

In the setting of hyponatremia, patients would gain sodium during dialysis as most dialysis fluid sodium concentration prescriptions range from 137 to 140 mmol/L. To avoid sodium gains due to diffusion, the concentration of sodium would need to be lower in the dialysis fluid than in the plasma water. One should also consider the fact that the serum aqueous sodium concentration is often higher than the total serum sodium concentration with dialysis fluid sodium concentration reflecting more the serum aqueous sodium concentration. It has been proposed to perform point-of-care determination of electrolyte concentrations prior to dialysis. The dialysis fluid sodium concentration can then be adjusted to allow for sodium removal. A four-stream bicarbonate-based fluid delivery system would allow adjusting sodium without changing bicarbonate concentration or the ingredients in the acid bath (93).

Determining plasma water sodium is complex. One must also account for sodium bound to protein and other ions in accordance to the Gibbs-Donnan phenomenon (89). Sodium modeling or profiling was developed to address symptomatic intradialytic hypotension. The theory behind sodium modeling explains that a high initial dialysis fluid sodium would offset the usual rapid decline in plasma sodium that occurs early in HD (due to rapid removal of solutes), thereby reducing osmotic gradients across cell membranes, improving vascular refill and reducing the fall in plasma volume (94). Sodium modeling or profiling provides saline at the beginning of dialysis to maintain intravascular volume which will prevent hypotension and cramping. The amount of sodium infused varies and depends on the sodium gradient between the prescribed dialysis fluid sodium and plasma water. Due to sodium gain resulting in thirst and an increase in IDWG, this process has gone out of favor.

Also of important consideration, is the fact that changes in the bicarbonate concentration in the dialysis fluid to correct acid–base disorders will result in a change in sodium concentration as the two are found in equal concentration in bicarbonate-based dialysis fluid (95, 96). Oral sodium bicarbonate has been prescribed to patients with end-stage kidney disease on dialysis to optimize pre-dialysis total carbon dioxide concentration and to, albeit unsuccessful, improve serum albumin levels (97–99).

During HD, sodium removal occurs mainly by convective processes, composed of UF ~78% and to a lesser degree by diffusion, ~22% if the sodium gradient is conductive (100). The amount of sodium removed by convection equals the plasma water sodium multiplied by the volume removed. Regarding diffusion, depending on the pre-dialysis plasma water and the dialysis fluid sodium concentration gradient there could be sodium gain (dialysis fluid sodium concentration > plasma water sodium concentration) or loss (dialysis fluid sodium concentration < plasma water sodium concentration). The rate of sodium removal from the intravascular compartment combined with the cardiovascular response determines the appearance of hypotension and cramps. Patients who can refill the intravascular volume with fluid from the interstitial and intracellular compartments experience no or fewer symptoms (101).

A higher dialysis fluid sodium concentration may alleviate disequilibrium symptoms and improve cardiovascular stability. Higher dialysis fluid sodium results in significant thirst, IDWG, and increased prevalence of hypertension. An excessive sodium load increases extracellular volume due to water shift from the intracellular space and thirst. This has been associated with a higher mortality risk (74).

Consequently, management systems minimizing the sodium flux into the patient utilizing physicochemical principles of electrolytes and their estimation using conductivity measurements, have been developed. These methods have been studied in various clinical settings and improvements of medium- and short-term outcomes of such sodium-centric dialysis individualization methods have been reported. While promising, the effects on long terms outcomes is yet to be studied in adequately powered, prospective research (102, 103).

#### Sources for sodium addition and removal in peritoneal dialysis

In PD, convection occurs in response to hyperosmotic PD fluid generated by varying concentration of dextrose. Due to the gentle nature of ultrafiltration, patients receiving PD experience fewer episodes of hypotension and cramps compared to patients receiving HD. Additionally, the continuous nature of PD allows continuous sodium removal. PD patients with persistent volume overload have a 60% higher mortality risk (104). As noted also earlier (70), high sodium intake corresponds to direct toxicity on the peritoneal membrane, leading to chronic inflammation, fibrosis, and hyper vascularization, i.e., high transport state (70). In addition, Gong and co-investigators reported an association of high sodium intake with higher decline in residual renal function in PD patients (105). PD patients treated with a cycler have a lower sodium removal because of the greater sodium sieving as compared to those on continuous ambulatory peritoneal dialysis (CAPD) (106).

### Dialysis fluid sodium in intensive hemodialysis

Dialysis fluid sodium level is one of the determinants of IDWG in HD patients. Use of higher dialysis fluid sodium concentrations may reduce the risk of intradialytic hypotension but may increase IDWG because of positive sodium balance during the HD session. Theoretically, this effect may be pronounced in more frequent and/or longer HD. It is not clear what the optimal dialysis fluid sodium concentration should be in intensive HD regimens (defined as daily nocturnal HD or frequent HD).

In HD patients, extended session duration offers lower UF rate, which reduces the frequency of intradialytic hypotensive episodes and facilitates euvolemia. Indeed, better BP control together with reduced antihypertensive medication requirement and improvement in several cardiac parameters have been repeatedly demonstrated in patients treated with intensive HD. On the other hand, an increase in IDWG has often been reported in patients who switched to nocturnal HD. Although this increase has been attributed to improved nutrition and/or more liberal fluid intake, the possible role of the sodium concentration in the dialysis fluid has not been adequately studied.

In the London Daily/Nocturnal Hemodialysis Study which included a group of patients receiving quotidian HD either short daily or long nocturnal and followed for 18 months, a standard dialysis fluid of 140 mmol/L was used (107), which is still lower than the serum aqueous sodium concentration of around 154 mmol/L. The predialysis mean arterial BP and the number of prescribed antihypertensive medications diminished in both daily and nocturnal HD. The IDWG and ECV significantly decreased in daily HD whereas the nocturnal HD group had transient but significant increase in IDWG at 6 and 15 months and no difference in ECV compared with controls (108).

In a retrospective case–control study involving thrice-weekly in-center nocturnal HD and standard of care HD patients from the US, it was shown that in-center nocturnal HD patients had greater IDWG, but lower BP levels compared to the standard of care HD patients (109). IDWG was 4.0 kg in nocturnal HD and 2.8 kg in the standard of care HD group. Mean SBP was lower by 2 mmHg before dialysis and by 5 mmHg after dialysis in the nocturnal HD group; data on antihypertensive medication and dialysis fluid concentration data was not available.

In a prospective case–control study comparing of 4- and 8-h dialysis session in prevalent HD patients, it was reported that left ventricular (LV) mass index, left atrial and LV end-diastolic diameters together with ejection fraction significantly improved in the nocturnal HD compared to the standard HD group at the end of 1 year follow-up (110). Dialysis fluid sodium concentration was 138 mmol/L in both groups. Despite no change in BP levels during follow up, the need for antihypertensive medication declined from 22 to 8% in nocturnal HD patients. IDWG was found to be higher in the nocturnal HD group (1.41 vs. 1.21 kg/day in nocturnal HD and standard HD) with the improvement of nutritional parameters. Despite this, the frequency of intradialytic hypotension markedly decreased in nocturnal HD compared to a slight increase in standard HD (110).

The Frequent Hemodialysis Network (FHN) Nocturnal Trial, which investigated the effect of frequent nocturnal home HD with standard home HD on composite outcomes had variable dialysis fluid sodium concentration with a mean of 139 ± 9 mmol/L (111). The patients treated with nocturnal HD had a 1.23-fold higher total weekly UF than standard home HD patients (9.1 L versus 7.4 L, respectively). The UF rate per session was 1.95 L in nocturnal HD compared to 2.52 L in standard home HD. As a secondary outcome, the mean difference in weekly average pre-dialysis SBP was −9.7 mmHg between groups (*p* < 0.01), whereas the mean difference in change in LV mass was only −5.2 g/m^2^ (95% CI, −11.4 to +1.0 g/m^2^) between the groups, favoring however more frequent HD in both outcomes.

It is possible to reach normovolemia/normal BP with longer HD sessions in addition to the implementation of salt restriction strategy as demonstrated by the Tassin group. The dialysis fluid sodium concentration was 138 mmol/L (29, 30).

Increasing frequency of in-center HD may result in beneficial changes in volume control. The FHN Daily Trial investigated the effect of six times a week HD on death or LV mass index for 12 months (112). The average number of sessions per week was 5.2 and the mean duration of each session was 154 min. Compared to the control group, the weekly UF rate was higher in daily HD (10.5 L vs. 8.9 L, *p* < 0.001). The mean difference in weekly average predialysis SBP was −10.1 mmHg between groups (*p* < 0.001) with a mean difference in change in LV mass of −13.8 g/m^2^ (95% CI, −21.8 to −5.8 g/m^2^, *p* < 0.001), in both cases favoring more frequent HD. Moreover, episodes of hypotension during dialysis were less common in the frequent HD group than in the control group (10.9% vs. 13.6%, *p* = 0.04). Although the dialysis fluid sodium concentration was not considered in the design of the trial, the treatment effect of frequent HD on LV mass was modified by serum sodium concentration (113). The reduction of LV mass was significantly higher in patients with a serum sodium concentration below 138 mmol/L (−28.0 g, 95%CI −40.5 to −15.4) than in patients with higher serum concentration (−2.0 g, 95%CI −15.5 to 11.5 g).

### Hemodiafiltration and sodium

#### HDF treatment and the association with a superior survival

In recent years, several studies have compared hemodiafiltration (HDF) with standard HD (41, 114–116). From a recent meta-analysis, it appeared that online post-dilution HDF is associated with a lower overall mortality than standard HD (all-cause mortality HR 0.86 [95% CI: 0.75; 0.99]). The largest reduction in mortality was achieved in patients receiving the highest convection volume (CV); > 23 L/1.73 m^2^/session (all-cause mortality HR 0.78 [95% CI: 0.62; 0.98]) (117). The CONVINCE study, an international, multi-center, prospective, randomized, controlled trial comparing high-dose HDF versus standard high-flux HD, addressing clinical endpoints, quality of life and a cost-utility analysis (118), has just recently concluded and corroborated the significant survival advantage conferred by HDF (119). The mechanism behind the suggested beneficial effect of HDF on mortality is not yet fully understood. Several mechanisms have been proposed, including increased toxin removal, improved hemodynamic stability and correction of sodium imbalance (120). Herein we solely focus on the topic of sodium balance during HDF.

#### HDF treatment and intra-dialytic hemodynamic stability

Several studies have shown an association between HDF and an improved intradialytic hemodynamic stability, when compared to standard HD (115, 121, 122). One possible explanation for this association is the difference in thermal balance between HDF and HD. It has been demonstrated that despite use of pre-warmed replacement fluid, online HDF results in cooling of the blood by enhanced thermal energy losses within the extracorporeal system (121). After correction for temperature, differences between HDF and HD on intradialytic hypotensive episodes were no longer observed (121, 123). Some studies have proposed reduced sodium removal during HDF as a mechanism to explain the improved hemodynamic stability (122, 124). Indeed, high sodium dialysis fluid concentrations are associated with improved hemodynamic stability on the short term (89). However, since a high dialysis fluid sodium concentration results in an increase in the IDWG and a rise in BP (90), obtaining hemodynamic stability by increasing sodium loading in HD or HDF is disadvantageous for the long-term. However, the data on sodium balance in HDF are not conclusive and are discussed below.

#### Effects of HDF treatment on sodium balance

In standard HD, diffusion and UF are the main determinants of the sodium balance. Additionally, intra-dialytic interventions including sodium containing fluid boluses to treat intradialytic hypotension, priming and rinsing of the extracorporeal circuit (88) also play an important role. In addition, the Gibbs-Donnan effect should be mentioned, implicating that sodium transport over the dialyzer membrane is generally lower than expected due to the negative charge of plasma proteins on the surface of the membrane (125).

In online post-dilution HDF, especially when a high CV is applied, a large amount of substitution fluid (which is manufactured online from the dialysis fluid and has the same electrolyte composition) is infused into the patient which can affect the sodium balance and consequently the fluid status. If there is a positive sodium gradient between the substitution fluid and the plasma, this will most likely result in net sodium retention and vice versa. However, the exact effects of the combination of diffusion and convection on sodium balance (as in HDF) are complex. In recent years, several studies have been conducted to investigate the effects of HDF on sodium balance.

In HDF, similar to standard HD, the post-dialysis sodium concentration in plasma water is slightly higher than the sodium concentration in the dialysis fluid **-** as explained by the Gibbs-Donnan effect (126). As this effect is largely dependent on the plasma protein concentration, it is conceivable that the Gibbs-Donnan effect is a much more important factor during post-dilution as compared to pre-dilution HDF. However, to the best of our knowledge this has not been investigated yet.

In the early 1990’s Pedrini et al. (127) performed a clinical validation of a computer simulated model on sodium and water kinetics during HDF (probably post-dilution with bags) and HD in 8 patients. They demonstrated that the sodium concentration in the substitution fluid was related to substantial changes in the sodium plasma water concentration. Especially, high UF rates during HDF resulted in sodium retention, as was explained by the Gibbs-Donnan effect. It was suggested that in order to maintain an adequate sodium balance, this effect should be counterbalanced by increased sodium removal by diffusion.

In another trial, the effects on sodium transport in 9 patients undergoing pre-dilution HDF were investigated. Treatment with pre-dilution HDF (mean sodium concentration substitution fluid was 141.6 mmol/L) resulted in more or less the same reduction of intradialytic sodium removal as in HD (124). Notably, in these “older” studies the sodium concentration of the substitution fluid was well above the sodium concentration currently applied in clinical practice. In more recent studies, as summarized below, lower sodium concentrations of the substitution fluid were used. These studies did not observe sodium retention when HDF was applied.

Locatelli et al. (122) randomized 146 dialysis patients to standard HD, online predilution hemofiltration (HF) or online pre-dilution HDF for 2 years. In accordance with previous studies, treatment with HDF and HF resulted in less intradialytic hypotension compared to standard HD, but no differences in the amount of sodium removal between HDF, HF and standard HD.

La Milia et al. (128) evaluated the sodium removal and plasma tonicity balance in a cross-over trial in which 47 patients were subjected to 2–3 consecutive sessions of high-flux standard HD followed by the same number of HDF sessions, or vice versa. Additionally, the mean sodium removal per dialysis session did not differ between high-flux HD and HDF, nor did the plasma tonicity. The magnitude of the convective volume was, however, not disclosed.

In a cross-sectional retrospective analysis, Chazot et al. (129) compared the fluid status of 2,242 dialysis patients treated with standard HD and online post-dilution HDF for 1 month. The pre-dialysis relative fluid overload status was assessed by using Body Compositor Monitor (BCM) measurements. The dialysis fluid sodium concentration was fixed at 140 mmol/L. Plasma sodium concentration was estimated from the dialysis fluid conductivity as monitored continuously by the dialysis machine. Among 694 HDF patients, no differences were found in the IDWG, the dialysis fluid to plasma sodium gradient and the pre-dialysis relative fluid overload as compared to pair matched HD controls. In addition, no association was found between HDF treatment and markers of fluid volume excess.

Finally, in a recent cross-over trial by Rodriguez et al. (130), 10 chronic dialysis patients were subjected to high-flux HD and high-volume (median convective volume was 21.5 L per HDF session) online post-dilution HDF during 4 phases, lasting 1 month each, with alternate use of HD and HDF. A new approach to calculate sodium mass removal using the ionic dialysance sensor embedded in the dialysis machine was used and compared to conventional methods to assess the total body water. The dialysis fluid sodium concentration was fixed at 138 mmol/L. With the new approach, a minimal difference in sodium mass transfer was observed between standard HD and HDF. Most interestingly, sodium mass removal in high-volume HDF was almost like standard HD. Moreover, the cumulative net ionic mass balance on a weekly basis was similar in HD and HDF. It was concluded that further studies are needed to be performed to evaluate whether the improved hemodynamic stability associated with online HDF was due to substitution fluid having a relatively higher hypertonic solution or a reduced net sodium mass balance must be ruled out.

To date, only a limited number of clinical studies have directly compared the effect of HDF on intra-dialytic sodium removal to standard HD (Table 3). Overall, the results from these studies are inconclusive. Factors that should be considered when comparing these studies are the sodium concentration of the substitution fluid (generally higher in the older studies) and the HDF modality (the Gibbs-Donnan effect is probably more prominent in post-dilution as compared to pre-dilution HDF). The dialyzer specifications could also be of importance, but this has not yet been studied. Since dialyzers that are specifically designed for HDF treatment have a different pressure profile, the Gibbs-Donnan effects will most likely be reduced.

**Table 3:** Effect of HDF compared with HD treatment on intra-dialytic sodium balance.

	Study design and population	Sodium concentration substitution fluid (mmol/L)	HDF modality	Sodium removal HDF vs. HD*	Effects on IDWG	Effects on pre- and post-dialysis BP
Pedrini et al. (127)	Computer model and clinical trial, Italy, *N* = 12	140–145	Not reported (probably post-dilution with bags)	↑	N/A	N/A
Di Filippo et al. (124)	Clinical trial, Italy, *N* = 9	141.6 ± 1.8	Post-dilution^#^	↑	N/A	N/A
Locatelli et al. (122)	RCT, Italy, *N* = 146	133–152	Pre-dilution	↔	Same amount of net UF volume in HDF as in HD.	+ 4.2 mmHg in mean pre-dialysis BP in HDF compared with HD.
La Milia et al. (128)	Cross-over trial, Italy, *N* = 47	138 (IQR 136–140)	Not reported	↔	N/A	No differences between HDF and HD.
Chazot et al. (129)	Cross-sectional retrospective analysis, *N* = 2,242	140 (IQR 139–140)	Post-dilution	↔	No differences between HDF and HD.	No differences in pre-dialysis SBP between HDF and HD. Post-dialysis BP N/A.
Rodriguez et al. (130)	Cross-over trial, France, *N* = 10	138 (range 138–140)	60% post-dilution	↔	Higher IDWG in HDF (2.0 kg) compared to HD (1.8 kg)	N/A

^*^Intradialytic sodium removal or effect on sodium balance by HDF as compared to standard HD. ^#^Hemofiltration. BP, blood pressure; HD, standard HD; HDF, hemodiafiltration; IDWG, interdialytic weight gain; UF, ultrafiltration; RCT, randomized controlled trial.

The most recent and methodologically best-designed studies did not show clinically significant differences in the intra-dialytic sodium removal between online (high-volume) post-dilution HDF and standard HD. Hence, we conclude with some uncertainty that sodium retention is not responsible for the increased hemodynamic stability in online post-dilution HDF.

## Conclusion

Sodium restriction continues to be a problematic topic in dialysis patients. On several levels dialysis patients are subjected to a positive sodium balance—either by dietary or dialytic means. While the understanding of both dimensions improves and active initiatives aim to improve and ameliorate patient outcomes, problems persist and areas for improvement are noted.

## Author contributions

SL: Conceptualization, Writing – original draft, Writing – review & editing. GA: Conceptualization, Writing – original draft, Writing – review & editing. PR: Conceptualization, Writing – original draft, Writing – review & editing. EO: Conceptualization, Writing – original draft, Writing – review & editing. EP: Conceptualization, Writing – original draft, Writing – review & editing. AT: Conceptualization, Writing – original draft, Writing – review & editing. TI: Conceptualization, Writing – original draft, Writing – review & editing. JR: Conceptualization, Writing – original draft, Writing – review & editing. RS: Writing – review & editing.
